# Exploring the efficacy of ^18^F-FDG PET/CT in hepatocellular carcinoma diagnosis: role of Ki-67 index and tumor differentiation

**DOI:** 10.1007/s00261-023-04027-4

**Published:** 2023-09-08

**Authors:** Yuping Yin, Jiachen Liu, Runlu Sun, Xuming Liu, Zhangchi Zhou, Hong Zhang, Dan Li

**Affiliations:** 1 Department of Nuclear Medicine, Sun Yat-sen Memorial Hospital, Sun Yat-sen University, Guangzhou, China; 2 Department of Cardiology, Sun Yat-sen Memorial Hospital, Sun Yat-sen University, Guangzhou, China; 3 Department of Pathology, Sun Yat-sen Memorial Hospital, Sun Yat-sen University, Guangzhou, China; 4 Department of Nuclear Medicine, Sun Yat-sen Memorial Hospital, No. 107, The West of Yanjiang Road, Guangzhou, 510120 China

**Keywords:** ^18^F-FDG PET/CT, PET/CT, Hepatocellular carcinoma, FDG-avidity, Ki-67 index

## Abstract

**Purpose:**

The sensitivity of [^18^F] fluorodeoxyglucose positron emission tomography-computed tomography (^18^F-FDG PET/CT) for detecting hepatocellular carcinoma (HCC) has not been clarified thoroughly. Our study seeks to explore the association between the Ki-67 index and FDG-avidity in HCC tumors using ^18^F-FDG PET/CT.

**Methods:**

112 HCC lesions from 109 patients detected by ^18^F-FDG PET/CT were included retrospectively between August 2017 and May 2022, comprising 82 lesions in the training cohort and 30 in the validation cohort to simulate prospective studies. In the training cohort, lesions were stratified by a lesion-to-liver maximum standardized uptake value (SUV_max_) ratio cut-off of 1.59. The relationships between lesion-to-liver SUV_max_ ratios and several clinical factors including tumor differentiation, alpha fetoprotein (AFP), carcinoembryonic antigen (CEA), hepatitis B virus (HBV) infection, Ki-67 index et al. were assessed. These findings were subsequently validated in the independent validation cohort.

**Results:**

In the training cohort, group A1 lesions demonstrated a higher Ki-67 index (%, 40.00 [30.00, 57.50] vs. 10.00 [5.00, 28.75], *p*<0.001) than group A0, the positive correlation between FDG-avidity and Ki-67 index was revealed by multivariate analysis, OR=1.040, 95% CI of OR [1.004–1.077], *p*=0.030. The calculated cut-off value was 17.5% using the receiver operating characteristic (ROC) curve, with an area under curve (AUC) of 0.834 and 95% CI [0.742–0.926], *p*<0.001. These findings were further validated in the independent validation cohort, with similar results (AUC=0.875, 95% CI [0.750–1.000], *p*<0.001).

**Conclusion:**

In comparison to tumor differentiation, Ki-67 index demonstrates a stronger association with FDG-avidity in HCC tumors, and when the Ki-67 index exceeds 17.5%, ^18^F-FDG PET/CT might serve as a useful indicator for HCC.

**Graphical abstract:**

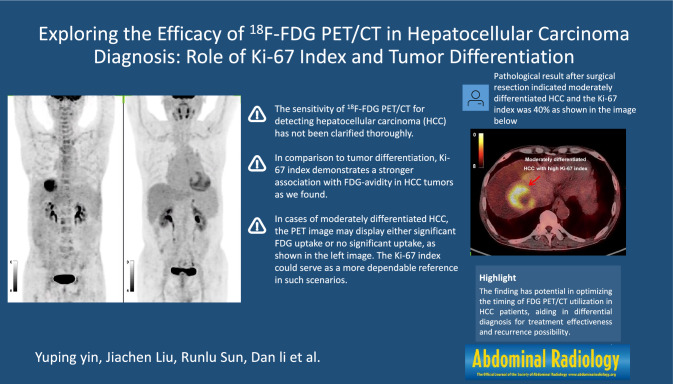

## Introduction

Recently, the International Agency for Research on Cancer (IARC) of the World Health Organization released the latest global cancer burden data for 2020, nearly 830,000 died of liver cancer, accounting for the third of all cancers, almost 90% of them were hepatocellular carcinoma, HCC [1]. Imaging techniques were a key tool in the diagnosis and monitoring of HCC. Non-invasive diagnostic criteria for HCC based on the imaging results of dynamic nuclear magnetic resonance imaging (MRI) and/or dynamic computed tomography (CT) had entered the guidelines and were widely used. [^18^F] fluorodeoxyglucose (^18^F-FDG) positron emission tomography-computed tomography (PET/CT) also plays an important role in the management of HCC due to its ability to show the metabolism of the lesion [2].

CT and MRI are capable of providing only observations on the morphological characteristics of HCC lesions [2], and the accuracy might be affected when the patient has a combination of liver cirrhosis [3, 4], at this point, the value of PET/CT to show the metabolism of the lesion begins to emerge. Although ^18^F-FDG PET/CT might have an important effect, the timing of its selection has not been well guided [5]. National Comprehensive Cancer Network (NCCN) guidelines (Version 1.2022) mentioned that “^18^F-FDG PET/CT has limited sensitivity but high specificity.” The main reason would be the low uptake of FDG by some HCC tumors [6]. A high expression of glucose-6-phosphatase [7] and low expression of GLUT1, GLUT3 [8] are some of the reasons associated with low FDG uptake in well-differentiated HCC. But to date, few studies have been done on the uptake ability of ^18^F-FDG in moderately differentiated HCC (accounting for a considerable proportion of HCC) [9]. Ki-67 is a well-known proliferation marker for the evaluation of cell proliferation, many previous studies had suggested that Ki-67 independently predicts cancer progression [10], and a study by Kitamura et al. mentioned that FDG uptake was also higher in Ki-67-positive lesions [11], but it is unclear whether the Ki-67 index (the percent positive frequency of Ki-67 protein labeling in HCC tumors) in HCC is useful in the interpretation of ^18^F-FDG PET/CT.

Therefore, this study aimed at the relation between the Ki-67 index and the ^18^F-FDG-avidity of HCC tumors.

## Materials and methods

### Study design

**Fig. 1:**
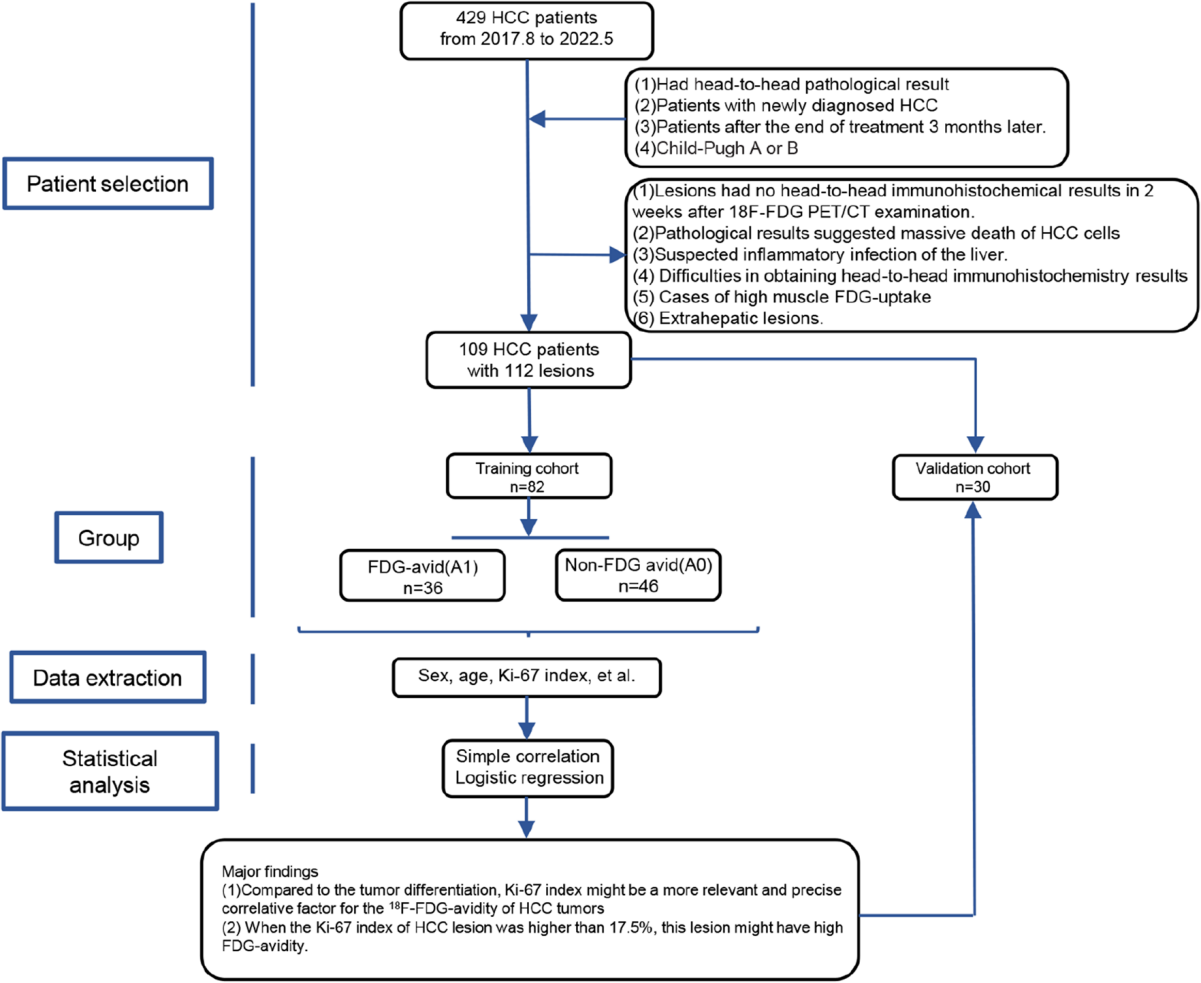
Workflow for this study design. *HCC* hepatocellular carcinoma, *HePpar-1* hepatocyte paraffin 1, *A1* HCC patients with FDG-avid lesions, *A0* HCC patients with non-FDG-avid lesions

This was a single-center retrospective cohort study with prospective content. Overall research workflow was depicted in Fig. 1, a brief description is as follows. Twenty-nine patients with 30 lesions were selected for validation cohort by computer-generated random numbers, the other 80 patients with 82 lesions were then allocated to the training cohort. The selected lesions in training cohort were divided into two groups visually firstly, according to the ^18^F-FDG PET/CT images, the prominent lesions were assigned to P1 (*n*=35), while the none prominent lesions were assigned to P0 (*n*=47), collecting data on metabolic tumor volume (MTV), total lesion glycolysis (TLG), the lesion-to-liver ratio [maximum standardized uptake value (SUV_max_), mean standardized uptake value (SUV_mean_)], and the lesion-to-blood pool ratio (SUV_max_, SUV_mean_), subsequently the lesion-to-liver SUV_max_ ratio equal to 1.59 was determined as the optimal cut-off point by receiver operating characteristic (ROC) curve, lesions with the ratio greater than or equal to 1.59 were assigned to FDG-avid group (A1, *n*=36), while the other were assigned to A0 (*n*=46). Age, tumor differentiation, alpha fetoprotein (AFP), carcinoembryonic antigen (CEA), hepatitis B virus (HBV) infection, Ki-67 index, and hepatocyte paraffin 1 (HePpar-1) status were extracted, next the simple correlation and logistical regression analysis were used to detect the association between them. Finally, we draw a conclusion that Ki-67 index was positively correlated with FDG-avidity in training and validation cohort, expectantly, the sensitivity of ^18^F-FDG PET/CT examination increased significantly when the Ki-67 index was above 17.5%.

### Patients

Between August 2017 and May 2022, 109 HCC patients with 112 tumor lesions (in 3 patients, two lesions each were selected for study) were selected from 429 patients with HCC who were detected by ^18^F-FDG PET/CT in our institution, and the patient demographics are shown in Tables 1 and 3. The inclusion criteria and exclusion criteria are as follows:

**Table 1:** Descriptive characteristics of included HCC patients in training cohort

Program	Training cohort	Validation cohort	*p*
Sex			
Male	72 (90%)	21 (72.41%)	0.022*
Female	8 (10%)	8 (27.59%)	
Age (years)	54.44±10.96	53.14±14.02	0.621
Differentiation			0.127
Poorly	12 (14.63%)	6 (20%)	
Moderately	52 (63.41%)	23 (76.67%)	
Well	11 (13.41%)	1 (3.33%)	
Null	7 (8.54%)	0	
AFP (ng/ml)	9.57 (4.10, 429.70)	163.10 (10.16, 3587.50)	0.043*
CEA (ng/ml)	2.90 (1.90, 4.60)	2.30 (1.70, 3.60)	0.185
HBV infection			0.704
Absent	14 (17.5%)	6 (20.69%)	
Present	66 (82.5%)	23 (79.31%)	
Ki-67 index (%)	25 (10, 43.75)	36.07±23.61	0.157
HePpar-1 status			0.237
Negative	9 (10.98%)	1 (3.33%)	
Low expression	5 (6.1%)	3 (10%)	
Medium expression	12 (14.63%)	8 (26.67%)	
Positive	51 (62.2%)	18 (60%)	
Null	5 (6.1%)	0	

Normally distributed continuous variables are presented as the mean ± standard deviation, while non-normally distributed data are presented as the median (IQR, interquartile range). Categorical variables are presented as the number (percentage)

*HCC* hepatocellular carcinoma, *AFP* alpha fetoprotein, *HBV* hepatitis B virus, *CEA* carcinoembryonic antigen, *HePpar-1* hepatocyte paraffin 1, *A1* HCC patients with FDG-avid lesions, *A0* HCC patients with non-FDG-avid lesions

*p < 0.05 and the symbol [italics] were considered significant

Inclusion criteria: (1) head-to-head pathological result confirmed that all the lesions were active HCC; (2) Patients with newly diagnosed HCC; and (3) Child–Pugh A or B.

Exclusion criteria: (1) lesions had not been surgically removed within 2 weeks after ^18^F-FDG PET/CT examination; (2) Specimens were too fragmented to obtain head-to-head pathology; (3) HCC lesions influenced by treatment interventions; (4) Extrahepatic lesions; and (5) Cases of high muscle FDG uptake due to poor preparation before PET/CT inspection.

The selected 109 HCC patients were divided into two cohorts, 29 patients with 30 lesions were selected for validation cohort by computer-generated random numbers, the other 80 patients with 82 lesions were allocated to the training cohort.

### Scan parameters

Instrument model: Germany Siemens Biograph m CT·S. CT scan parameters: CT tube pressure 120 kV, tube current: 300 MA, layer thickness 3–4 mm, matrix 512×512, radiopharmaceutical ^18^F-FDG, generated by GE800trace accelerator, imaging agent quality control radiochemical purity> 95%. ^18^F-FDG was injected intravenously at 5.55 MBq/kg body weight. After the drug was injected, the patient should close his(her) eyes and rest quietly for 40–60 min. PET/CT scan was performed after urination. Scan time 15 min. The obtained images were transferred to the Medix workstation for reading and analysis after attenuation correction processing [12].

### Results of PET/CT evaluation

PET/CT reports were written by a senior nuclear medicine physician working for more than 5 years, and reviewed by a nuclear medicine department associate chief physician. If the result was uncertain, the department would discuss it to make a conclusion, and the immunohistochemical results would not be disclosed. Final data collection was done by a third person. The SUV of detectable lesions was measured by drawing a region of interest (ROI) over the area of tumor, MTV and TLG were measured by Medix workstation with threshold 40%. The SUV of the liver was measured at the right lobe of liver in PET images by using a 10-mm round-shaped ROI, the SUV of the blood pool was measured in the lumen of the descending aorta in the flat main bronchial bifurcation.

### Results of pathology evaluation

The evaluation of immunohistochemical staining results was undertaken by a duo of experienced pathologists. Quantitative appraisals were consolidated and subsequently subjected to further scrutiny by a skilled pathologist. In situations where disparities emerged, an impartial third party was enlisted to undertake a comprehensive re-evaluation, including pathological reassessment. The preservation of research hypotheses' confidentiality was meticulously maintained throughout the assessment process, thereby upholding the principle of objectivity.

### Data collection

Traditional factors including age, sex (male or female), metabolic tumor volume (MTV), total lesion glycolysis (TLG), the lesion-to-liver ratio (SUV_max_, SUV_mean_) and the lesion-to-blood pool ratio (SUV_max_, SUV_mean_), tumor differentiation (divided into three gradients: poorly, moderately, well), AFP and CEA values in laboratory tests (normal reference range: AFP≤7 ng/ml, CEA≤5 ng/ml), HBV infection, Ki-67 index (the percentage of Ki-67 expression in HCC cells), HePpar-1 status (divided into four gradients: negative, low expression, medium expression, positive).

### Statistical analysis

Student's *t* test and Pearson test correlation test were used to test the differences in variables with normal distribution, and non-parametric tests (Kruskal–Wallis test, Mann–Whitney *U* test, and Spearman's rank correlation coefficient) were used for variables with non-normal distribution. The differences in categorical variables were tested by *χ*^2^ test and Fisher's test. All data of interest were then entered into the logistic regression equation for correction in training cohort. Finally, ROC curve was used to evaluate the predictive value. The two-side *p* <0.05 was considered significant. All statistical data were analyzed using SPSS 25 statistical software.

## Results

### Selection of the optimal cut-off point for whether a lesion is FDG-avid or not

**Fig. 2:**
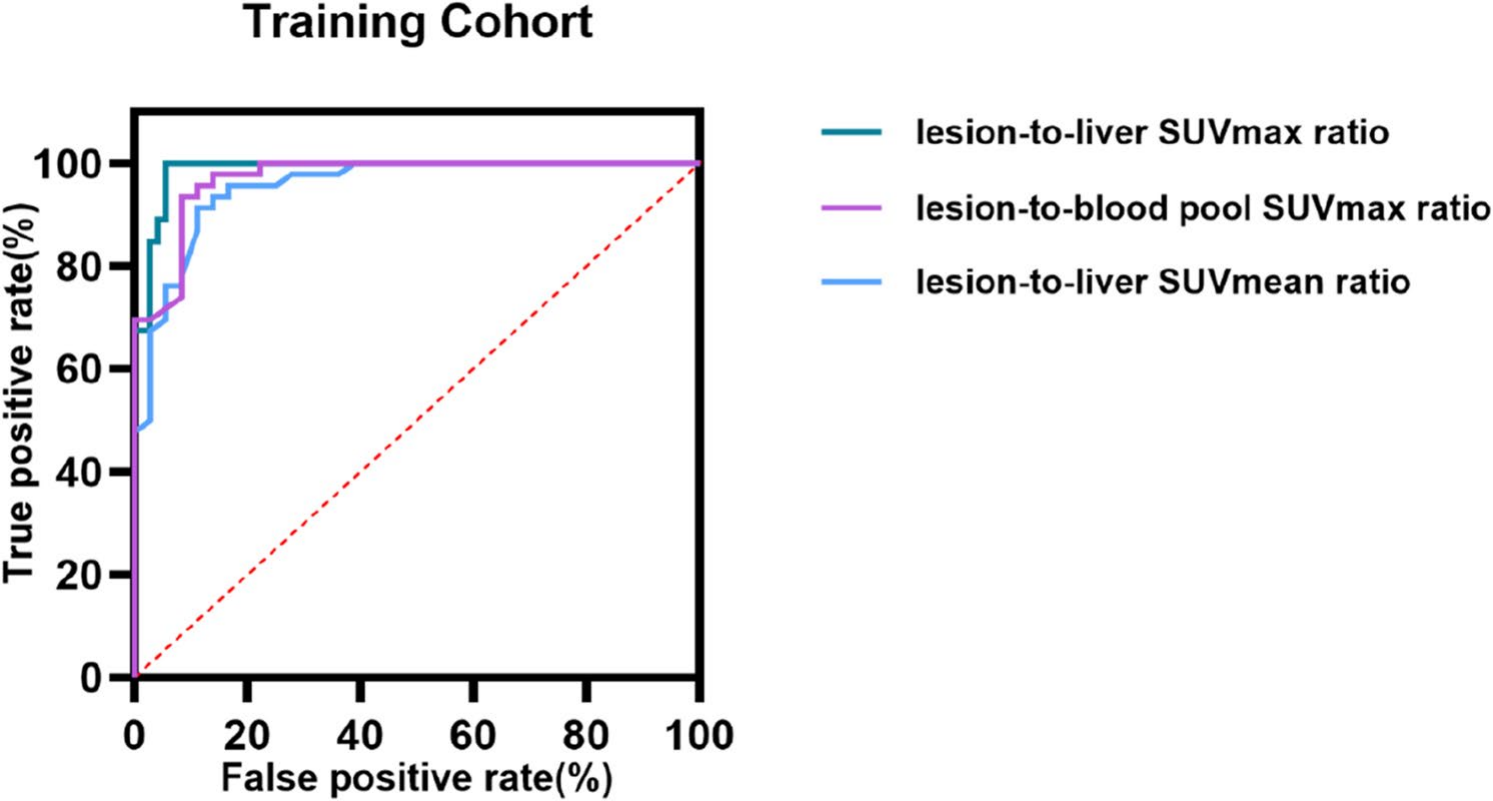
Lesion characteristics regarding FDG uptake predicting grouping visually in training cohort. ROC curve for lesion-to-live SUV_max_ ratio (AUC=0.987, *p* < 0.01, 95% CI [0.967–1.000]), lesion-to-live SUV_mean_ ratio predicting grouping (AUC=0.955, *p* < 0.01, 95% CI [0.914–0.996]), and lesion-to-blood pool SUV_max_ ratio (AUC=0.971, *p* < 0.01, 95% CI [0.941–1.000]) predicting grouping. *ROC* receiver operating characteristic, *AUC* area under curve, *CI* confidence interval. *p* < 0.05 was considered significant

Patient characteristics of training and invalidation cohort are given in Table 1. Lesion characteristics regarding FDG uptake of training cohort are given in Table 2. The lesion-to-liver ratio (SUV_max_, SUV_mean_) in P1 group was higher than P0 group, as was the lesion-to-blood pool ratio (SUV_max_). No significant difference in the lesion-to-blood pool ratio (SUV_mean_), MTV and TLG between the two groups. The ROC curve suggested that the lesion-to-liver ratio (SUV_max_) was the variable with the highest predictive grouping value as shown in Fig. 2, 1.59 was the optimal cut-off point calculated by Youden index, lesions with the ratio greater than or equal to 1.59 were regarded as FDG-avid lesions and grouped by A1, the other were regarded as none FDG-avid lesions and grouped by A0.

**Table 2:** Lesion characteristics regarding FDG uptake of training cohort

	P1, *n*=35	P0, *n*=47	*p*
Lesion-to-liver ratio			
SUV_max_	2.22 (1.82, 3.21)	1.16±0.22	< 0.001*
SUV_mean_	1.73 (1.39, 2.23)	0.91 (0.84, 1.08)	< 0.001*
Lesion-to-blood pool ratio			
SUV_max_	4.10 (3.47, 6.94)	1.94±0.48	< 0.001*
SUV_mean_	1.73 (1.39, 2.23)	1.53 (1.35, 1.81)	0.108
MTV (cm^3^)	6.62 (2.84, 32.49)	17.07 (8.01, 27.83)	0.095
TLG (cm^3^)	43.98 (14.27, 173.22)	47.13 (20.88, 83.09)	0.591

Normally distributed continuous variables are presented as the mean ± standard deviation, while non-normally distributed data are presented as the median (IQR, interquartile range)

*P1* patients with prominent HCC lesions visually, *P0* patients with non-prominent HCC lesions visually, *MTV* metabolic tumor volume, *TLG* total lesion glycolysis

*p < 0.05 and the symbol [italics] were considered significant

### Patient characteristics

A total of 82 lesions in 80 patients were included in training cohort, the HCC patients with FDG-avid lesions (A1) were younger than group A0 in Table 3, group A1 lesions had higher Ki-67 index than group A0, and so did AFP. The lesions in group A1 had worse differentiation compared to group A0. Sex, CEA, HBV infection, and HePpar-1 status were similar between A1 and A0 group, shown in Table 3.

**Table 3:** Comparison between patients with FDG-avid and non-FDG-avid lesions in training cohort

	A1, *n*=36	A0, *n*=46	*p*
Gender			
Man	33	41	1.000
Woman	3	5	
Age (years)	51.03±11.99	57.11±9.36	0.022*
Differentiation			< 0.001*
Poorly	10 (30.30%)	2 (4.35%)	
Moderately	23 (63.89%)	29 (63.04%)	
Well	1 (2.78%)	10 (21.74%)	
Null	2 (5.56%)	5 (10.87%)	
AFP (ng/ml)	54.80 (5.41, 1662.50)	6.42 (3.53, 25.90)	0.003*
CEA (ng/ml)	2.50 (1.83, 3.68)	3.50 (1.80, 5.60)	0.187
HBV infection			0.736
Absent	30 (83.33%)	37 (80.43%)	
Present	6 (16.67%)	9 (19.57%)	
Ki-67 Index (%)	40.00 (30.00, 57.50)	10.00 (5.00, 28.75)	< 0.001*
HePpar-1 status			0.168
Negative	7 (19.44%)	2 (4.88%)	
Low expression	3 (8.33%)	2 (4.88%)	
Medium expression	6 (16.67%)	6 (14.63%)	
Positive	20 (55.56%)	31 (75.61%)	

Normally distributed continuous variables are presented as the mean ± standard deviation, while non-normally distributed data are presented as the median (IQR, interquartile range). Categorical variables are presented as the number (percentage)

*SUV*_*max*_* ratio* lesion-to-liver SUV_max_ ratio, *AFP* alpha fetoprotein, *HBV* hepatitis B virus, *CEA* carcinoembryonic antigen, *HePpar-1* hepatocyte paraffin 1, *A1* HCC patients with FDG-avid lesions, *A0* HCC patients with non-FDG-avid lesions

*p < 0.05 and the symbol [italics] were considered significant

### Association between characteristics and FDG-avidity detected by ^18^F-FDG PET/CT inspection in training cohort

**Fig. 3:**
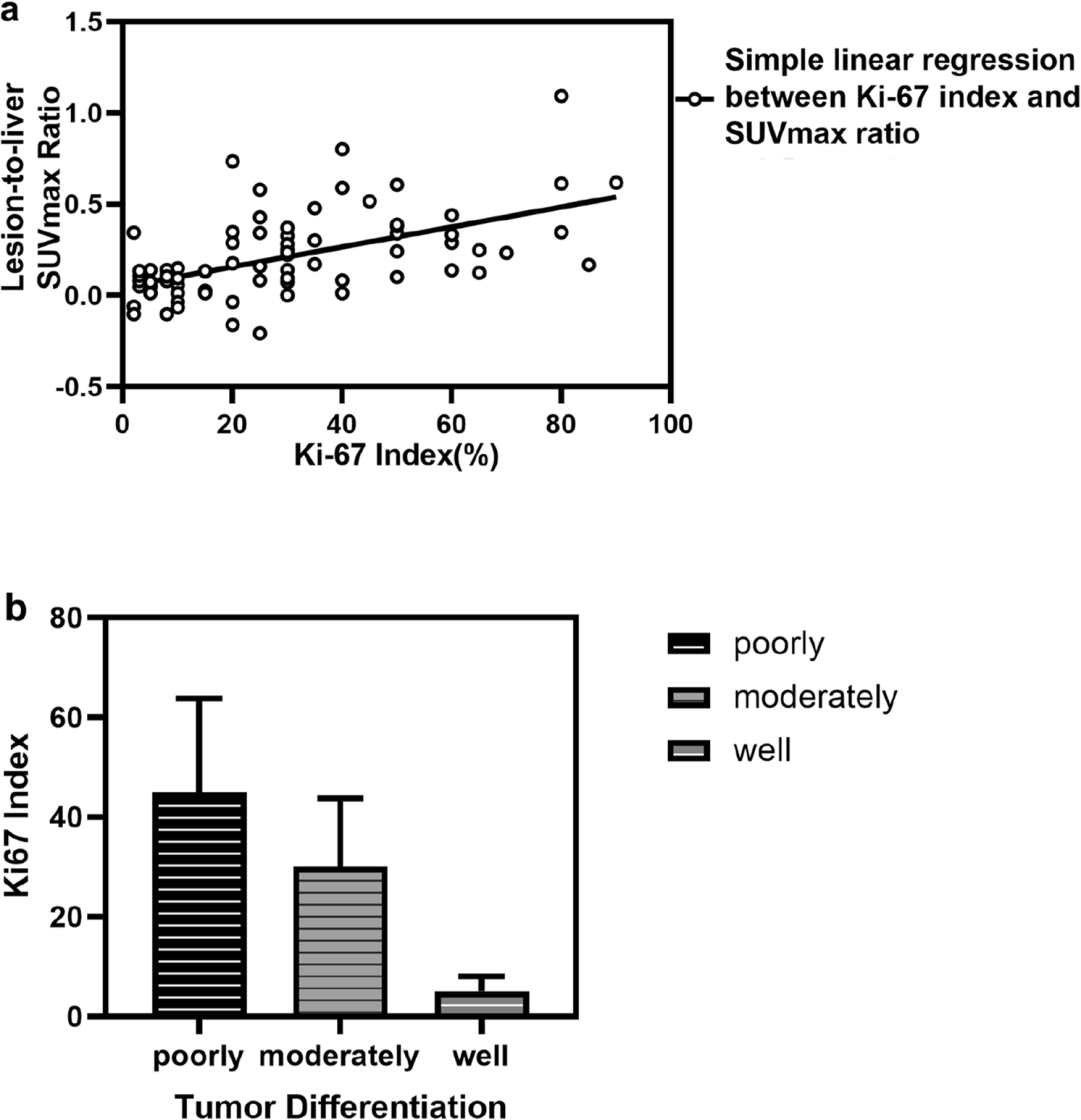
Correlations between Ki-67 index and lesion-to-liver SUV_max_ ratio, tumor differentiation in training cohort. **a** Simple linear regression between Ki-67 index and lesion-to-liver SUV_max_ ratio in training cohort, and *r*=0.563, *p* < 0.01. *SUV*_*max*_* ratio* lesion-to-liver SUV_max_ ratio; *p* < 0.05 was considered significant. **b** Distribution of Ki-67 index in HCC lesions with different degrees of differentiation. Ki-67 index was highest in poorly differentiated HCC lesions, followed by moderately differentiated and lowest in highly differentiated lesions, *p* < 0.05. *HCC* hepatocellular carcinoma

The associations between characteristics above and the FDG-avidity of lesions were analyzed by simple correlation. Data in Table 4 show that Ki-67 index was positively correlated with FDG-avidity, and so did AFP. While age, tumor differentiation, and HePpar-1 status were negatively correlated with it. The correlations above were tested by Spearman's rank correlation coefficient. The associations between Ki-67 index and lesion-to-liver SUV_max_ ratio are given in Fig. 3a by scatter plot, the associations between Ki-67 index and tumor differentiation are given in Fig. 3b.

**Table 4:** Association between characteristics and FDG-avid lesions in training cohort

	A	
	*r*	*p*
Age	− 0.236	0.033*
Tumor differentiation	− 0.520	< 0.001*
AFP	0.338	0.003*
CEA	− 0.162	0.189
Ki-67 index	0.538	< 0.001*
HePpar-1 status	− 0.256	0.025*

*AFP* alpha fetoprotein, *CEA* carcinoembryonic antigen, *HePpar-1* hepatocyte paraffin 1

*p < 0.05 and the symbol [italics] were considered significant

Next, characteristics that had a significant correlation (*p*<0.005) with FDG-avid lesions were entered as logistic regression, such as Ki-67 index, tumor differentiation, AFP and HePpar-1 status, conventional factors (age, sex), and MTV were used to correct. As shown in Table 5, only the Ki-67 index was positively correlated with FDG-avid lesion, and the tumor differentiation had no significant correlation with FDG-avidity after correction.

**Table 5:** Association between characteristics and FDG-avid lesions by logistic regression in training cohort

	*B*	SE	*p*	OR	95% CI of OR	
					Lower	Upper
Age	− 0.023	0.030	0.432	0.977	0.922	1.035
Sex	0.077	1.095	0.944	1.081	0.126	9.241
Differentiation	− 0.786	0.441	0.075	0.456	0.192	1.081
AFP	0.000	0.000	0.614	1.000	1.000	1.001
Ki-67	0.039	0.018	0.030*	1.040	1.004	1.077
HePpar-1	0.047	0.998	0.962	1.048	0.148	7.414

*AFP* alpha fetoprotein, *HePpar-1* hepatocyte paraffin 1

*p < 0.05 and the symbol [italics] were considered significant

**Fig. 4:**
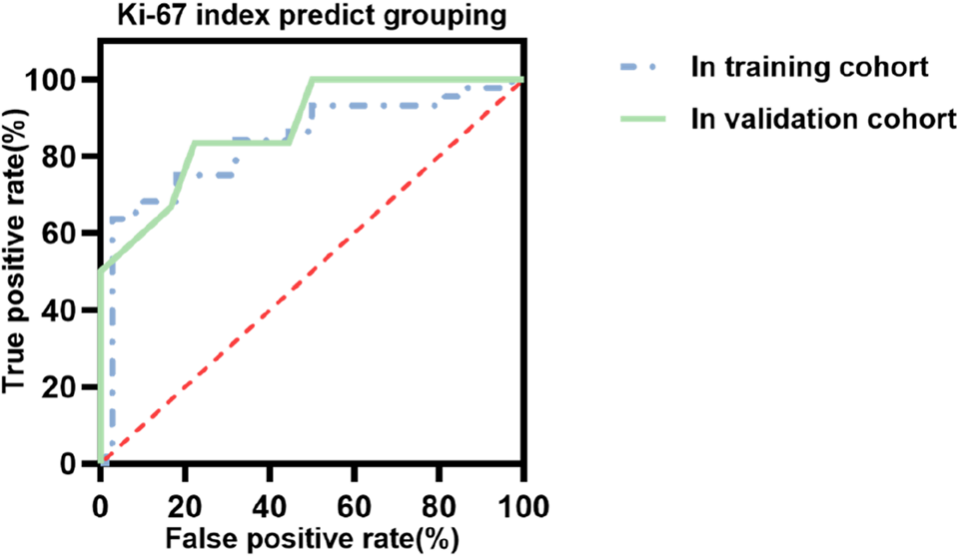
ROC curve for Ki-67 index predicting grouping in training and validation cohort. ROC curve for Ki-67 index predicting grouping in training cohort (AUC=0.834, *p* < 0.01, 95% CI [0.742–0.926]) and in validation cohort (AUC=0.875, *p* < 0.01, 95% CI [0.750–1.000]). *ROC* receiver operating characteristic, *AUC* area under curve, *CI* confidence interval. *p* < 0.05 was considered significant

Finally, ROC curve was used to evaluate predictive values of the Ki-67 index for grouping in training cohort as shown in Fig. 4a and Youden index indicated the cut-off value was 17.5%.

### The predictive value of Ki-67 index for FDG-avidity evaluated by ROC curve in validation cohort

There were 14 FDG-avid lesions and 16 non-FDG-avid lesions in validation cohort (*n*=30), the lesions were divided by lesion-to-liver SUV_max_ ratio, cut-off value was 1.59 (conclusion in Fig. 2a), FDG-avid lesions in validation cohort had higher Ki-67 index (%, 55.00 [40.00, 72.50] vs. 17.50 [11.50, 36.25], *p*<0.001) than non-FDG-avid lesions. ROC curve was used to evaluate predictive values as is shown in Fig. 4b, AUC=0.875, 95% CI [0.750–1.000], *p*<0.001. The validation cohort was divided into group 1 (HCC tumors with Ki-67 index above 17.5%, *n*=20, and the number of FDG-avid lesions was 14, the number of non-FDG-avid lesions was 6) and group 2 (HCC tumors with Ki-67 index below 17.5%, *n*=10, and the number of FDG-avid lesions was 0, the number of non-FDG-avid lesions was 10). Among the 82 lesions included in the training cohort, the sensitivities of ^18^F-FDG PET and ^18^F-FDG PET/CT were 41.25%, 68.75%. Among the 20 lesions in group 1 (HCC tumors with Ki-67 index above 17.5% in validation cohort), the sensitivities of ^18^F-FDG PET and ^18^F-FDG PET/CT were 70.00%, 80.00%, which were higher and *χ*^2^ test indicated *p*=0.036, while among the 10 lesions in group 2 (HCC tumors with Ki-67 index below 17.5% in validation cohort) the sensitivities were 0.00%, 20.00%, which were lower and Fisher's test indicated *p*=0.006.

### Examples

**Fig. 5:**
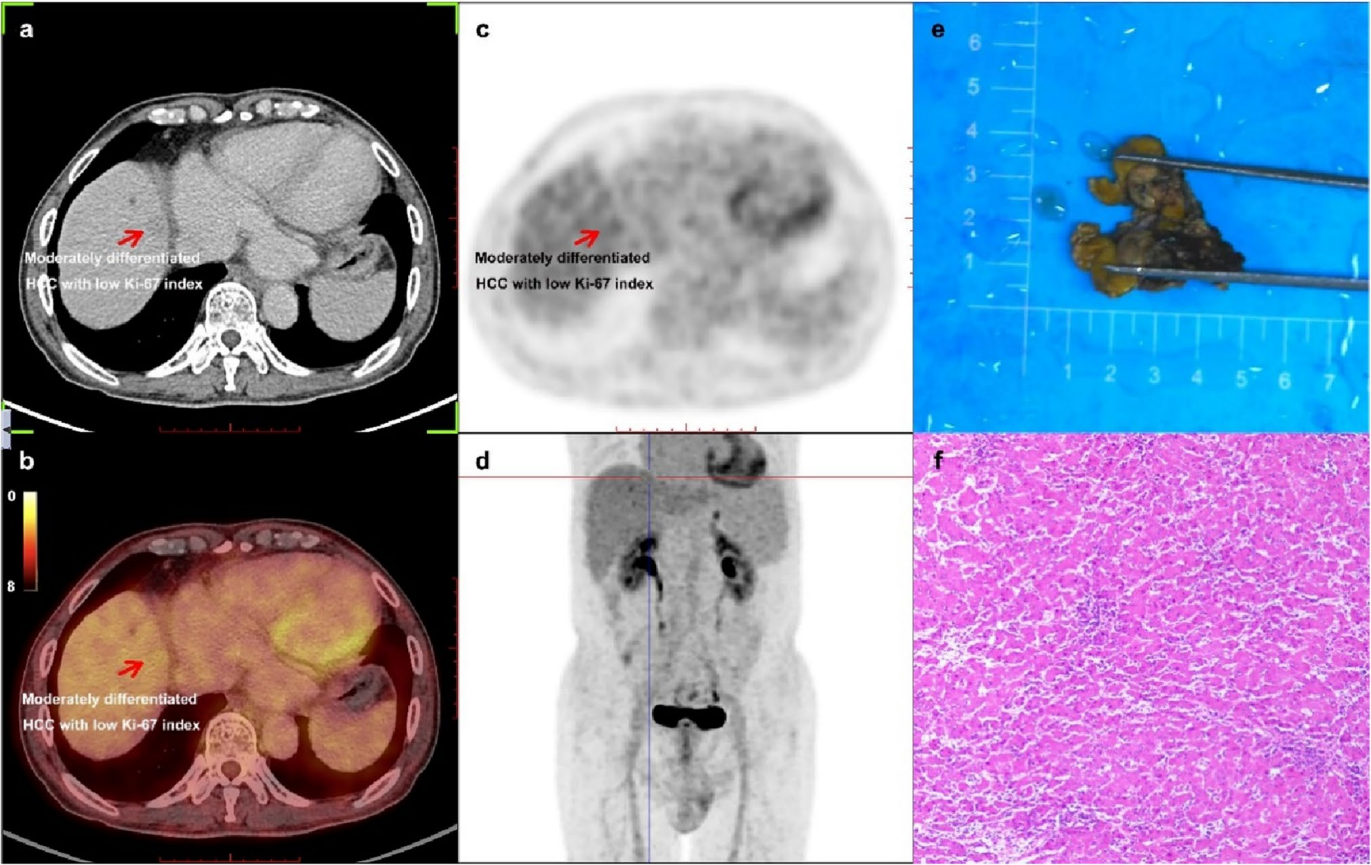
Example for a 70-year-old newly diagnosed HCC male man detected by ^18^F-FDG PET/CT inspection. **a** CT (computed tomography) image of the lesion located in liver S8 which was a hypodense nodule on delayed phase. **b** PET/CT fusion image of the lesion. **c** PET image of the lesion, and the SUV_max_ (maximum standardized uptake value) of this lesion was 3.10, the lesion-to-liver SUV_max_ ratio was 1.03. **d** The MIP (maximum intensity projection) picture of this patient.** e** Picture of the lesion after surgical resection.** f** A hematoxylin-stained pathological picture of this lesion indicated moderately differentiated HCC and the Ki-67 index was 10%. *HCC* hepatocellular carcinoma, ^*18*^*F-FDG PET/CT* [^18^F] fluorodeoxyglucose positron emission tomography-computed tomography

**Fig. 6:**
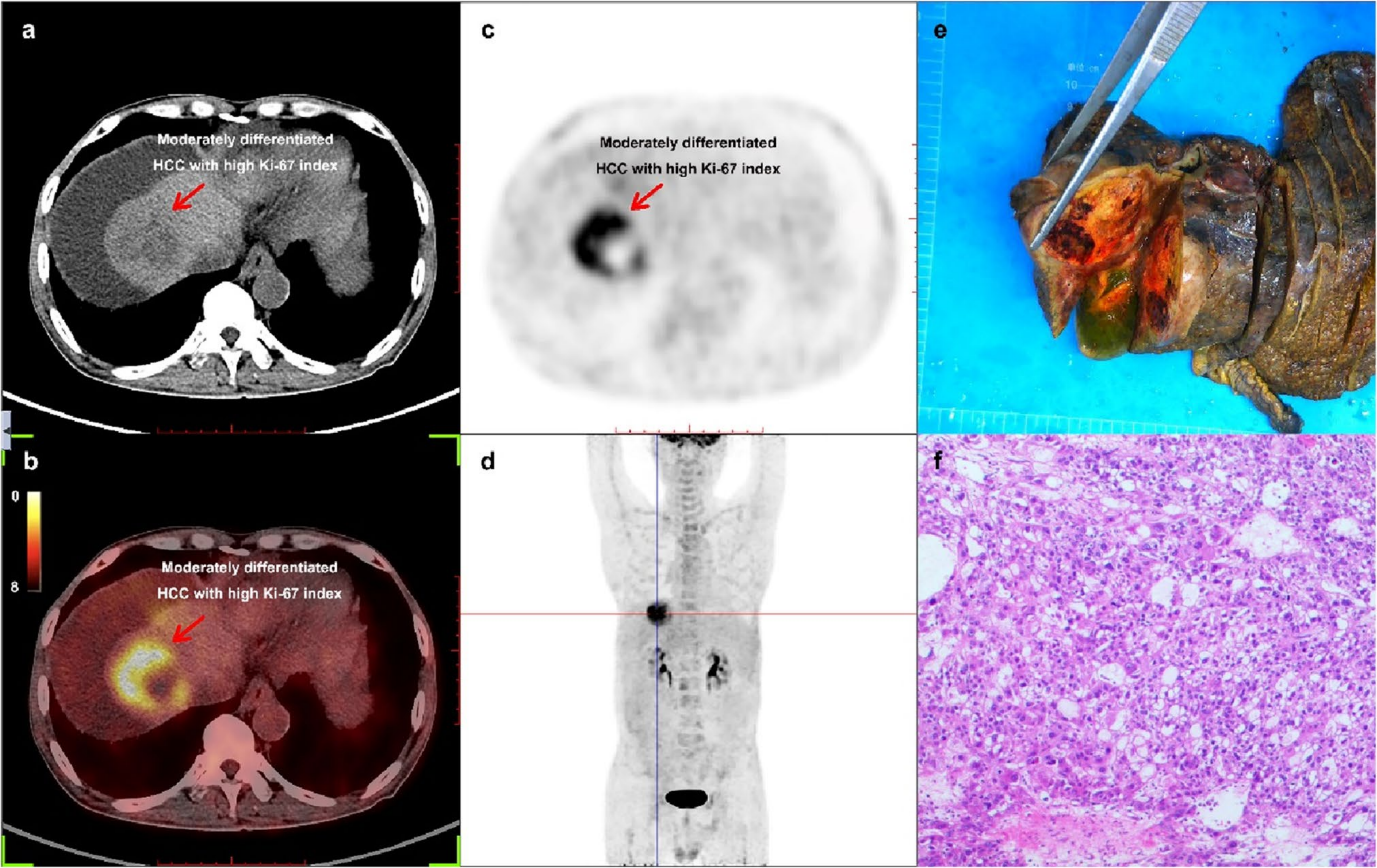
Example for a 66-year-old HCC male patient who was relapsed after interventional therapy and detected by ^18^F-FDG PET/CT inspection. **a** CT (computed tomography) image of the lesion located in liver S8 which was an inhomogeneous hypodense nodule. **b** PET/CT fusion image of the lesion. **c** PET image of the lesion, and the SUV_max_ (maximum standardized uptake value) of this lesion was 9.30, the lesion-to-liver SUV_max_ ratio was 3.88. **d** The MIP (maximum intensity projection) picture of this patient.** e** Picture of the lesion after surgical resection.** f** A hematoxylin-stained pathological picture of this lesion indicated moderately differentiated HCC and the Ki-67 index was 40%. *HCC* hepatocellular carcinoma, ^*18*^*F-FDG PET/CT* [^18^F] fluorodeoxyglucose positron emission tomography-computed tomography

Figure 5 shows an example of a 70-year-old newly diagnosed HCC male patient detected by ^18^F-FDG PET/CT inspection. The SUV_max_ of this lesion located in liver S8 was 3.10, while the SUV_max_ of normal liver parenchyma in right lobe of liver was 3.00, thus the lesion-to-liver SUV_max_ ratio was 1.03, pathological results after surgical resection indicated moderately differentiated HCC, and the Ki-67 index was 10%. Figure 6 shows another example of a 66-year-old HCC male patient who was relapsed after interventional therapy. As the result of ^18^F-FDG PET/CT inspection, the SUV_max_ of this lesion located in liver S8 was 9.30, while the SUV_max_ of normal liver parenchyma in right lobe of liver was 2.40, thus the lesion-to-liver SUV_max_ ratio was 3.88, pathological results after surgical resection indicated moderately differentiated HCC and the Ki-67 index was 40%.

These two examples indicated that the FDG-avidity could be high or low for moderately differentiated HCC, but it was still associated with Ki-67 index.

## Discussion

Intriguingly, it would be a novel strategy to help better application of ^18^F-FDG PET in the diagnosis and treatment of HCC patients based on the Ki-67 index. In this research, we found that (1) visual grouping and semi-quantitative analysis by lesion-to-liver SUV_max_ ratio were highly consistent (AUC=0.987, 95% CI [0.967–1.000], *p*<0.001), and cut-off value was 1.59 [13], lesions with the ratio greater than or equal to 1.59 were considered as FDG-avid (easy to be detected by ^18^F-FDG PET). (2) Tumor differentiation and HePpar-1 status were negatively correlated with the FDG-avidity of tumor lesion, while the Ki-67 index was positively correlated with it, but after correction, only the relationship between Ki-67 index and the FDG-avidity was significant. Compared to the tumor differentiation, Ki-67 index might be a more relevant and precise correlative factor for the ^18^F-FDG uptake of HCC tumors. (3) When the Ki-67 index of HCC lesion was more than 17.5%, this lesion might have higher FDG-avidity, the sensitivity of ^18^F-FDG PET/CT examination also increased significantly.

Many previous studies suggested that ^18^F-FDG PET/CT has low sensitivity in the diagnosis of HCC, the sensitivity was based on tumor cells uptake more ^18^F-FDG than surrounding [6, 14]. There were many reasons found recent years why the sensitivity was low in the diagnosis of HCC by ^18^F-FDG PET/CT, the most popular theory was that the low hexokinase activity and high glucose-6-phosphatase activity in well-differentiated HCC tumors, and resulting in the uptake of ^18^F-FDG by HCC lesion similar to that of normal liver parenchyma [7], however, this theory could not take into account the moderately differentiated HCC (accounts for a considerable proportion of HCC).

Ki-67 is well known as a kind of proliferative nuclear marker, which was first identified by Gerdes, etc. [15]. Ki-67 is a nuclear DNA binding protein, it is widely expressed in proliferating cells, many previous studies about cell cycle had shown that Ki-67 was present in the G_1_, S, and G_2_ phases of the cell cycle, but not in the G_0_ phase [10, 16], therefore, its high expression often represents that the proliferation of the cells is relatively vigorous. Many studies have shown that Ki-67 plays an important role in tumor grading and prognosis [17, 18], including HCC, studies had shown that high expression of Ki-67 predicted poorer prognosis [19], but its application in the interpretation of ^18^F-FDG PET/CT has not been studied. In this research, we found that Ki-67 index is positively correlated with the FDG-avidity of HCC lesion, and when an FDG-avid HCC lesion was found, the Ki-67 index might be more than 17.5% by immunohistochemistry. The reason might be the expression of glucose transporters (GLUTs). GLUT1 and GLUT3 are highly expressed in many tumors and both are thought to be highly correlated with the FDG-avidity of tumor [20, 21]. The expression of GLUTs protein is regulated by different signaling pathways and transcription factors in different tumors, however, in HCC tumors, the relationship between Ki-67 expression and GLUT1, GLUT3 protein expression is not clear until now [22].

In this study, we found that the FDG-avidity of HCC tumors have a correlation with the tumor differentiation, thus, poorly differentiated tumor with higher FDG-avidity and well-differentiated tumor with lower, which was also consistent with the classical views [22-26]. However, when the differentiation and the Ki-67 index entered the logistic regression equation together, only the correlation between Ki-67 index and FDG-avidity was significant. The reason might be that there is a correlation between Ki-67 index and differentiation of HCC tumors [19, 27], we also reached similar conclusions in this study, but Ki-67 index might be a more independent, relevant, and precise correlative factor for the ^18^F-FDG uptake of HCC tumors [28]. Another major reason might be that the FDG-avidity can be high or low for moderately differentiated HCC [7, 14, 29], as shown in Figs. 5 and 6.

When the Ki-67 index of HCC lesion was above 17.5%, this lesion might have high FDG-avidity. This might not only guide clinicians in the selection of ^18^F-FDG PET/CT examinations (because PET/CT is a radiological and high-cost examination [30]), but also play a role in image-guided treatment. For example, some targeted therapies targeting Ki-67 protein mainly guided by pathological biopsy results [10], however, the results of pathological biopsy might be affected by many conditions, such as biopsy site, section, and staining [31, 32], analyzing the FDG-avidity of the lesions might also help to guide the biopsy. When the Ki-67 index of HCC lesion was below 17.5%, other tumor imaging agents such as choline might need to be given more consideration [33]. The postoperative follow-up of HCC was often complicated by inflammation and bile leakage [34], and active foci of FDG metabolism in the hepatic region might be challenging to identify [35]. This study's findings might facilitate their detection.

Our study has some limitations. In the validation cohort group 1 (Ki-67 index above 17.5%), there were still 30% of patients with non-FDG-avid tumors, which might need to be combined with additional indicators for a more precise determination. Additionally, the attainment of a more precise Ki-67 index cut-off value requires a substantial sample size. These requirements necessitate further research efforts, and we are in the process of establishing prospective multi-center clinical trials to acquire more accurate data. At last, the intrinsic relationship between Ki-67 expression and FDG-avidity in HCC tumor remains unclear, these may require more laboratory research work by more people.

In conclusion, this study found a positive relationship between Ki-67 index and FDG-avidity in HCC tumors by ^18^F-FDG PET/CT that Ki-67 index might be a more effective predictor of ^18^F-FDG PET/CT sensitivity than tumor differentiation in HCC diagnosis and treatment. ^18^F-FDG PET/CT might be a good hint for HCC when Ki-67 index is higher than 17.5%, the sensitivity of ^18^F-FDG PET/CT examination increased significantly at this time. This might not only guide clinicians in the selection of ^18^F-FDG PET/CT examination, but also might provide a new way in image-guided treatment, and this discovery has the potential to assist nuclear medicine physicians in effectively utilizing FDG PET/CT for differential diagnosis of HCC concerning treatment effectiveness and the possibility of recurrence.

## Data Availability

All data were transparent. The data used in the current study are available from the corresponding authors on reasonable request.
